# Internal Validity of Two Promising Methods of Altering Temporal Orientation among Cigarette Smokers

**DOI:** 10.3390/ijerph182312601

**Published:** 2021-11-29

**Authors:** Richard J. O’Connor, Ellen Carl, Alina Shevorykin, Jeffrey S. Stein, Darian Vantucci, Amylynn Liskiewicz, Lindsey Bensch, Hannah Thorner, Matthew Marion, Andrew Hyland, Christine E. Sheffer

**Affiliations:** 1 Roswell Park Comprehensive Cancer Center, Buffalo, NY 14203, USA; richard.oconnor@roswellpark.org (R.J.O.); alina.shevorykin@roswellpark.org (A.S.); darian.vantucci@roswellpark.org (D.V.); amylynn.liskiewicz@roswellpark.org (A.L.); lindsey.bensch@roswellpark.org (L.B.); hannah.thorner@roswellpark.org (H.T.); matthew.marion@roswellpark.org (M.M.); andrew.hyland@roswellpark.org (A.H.); christine.sheffer@roswellpark.org (C.E.S.); 2 Center for Transformative Research on Health Behaviors, Fralin Biomedical Research Institute at VTC, 1 Riverside Circle, Roanoke, VA 24016, USA; jstein1@vtc.vt.edu

**Keywords:** episodic future thinking, future thinking priming, linguistic analysis, internal validity, temporal orientation, goal-attainment, smoking cessation

## Abstract

Relapse to smoking continues to be among the most urgent global health concerns. Novel, accessible, and minimally invasive treatments to aid in smoking cessation are likely to improve the reach and efficacy of smoking cessation treatment. Encouraging prospection by decreasing delay discounting (DD) is a new therapeutic target in the treatment of smoking cessation. Two early-stage interventions, delivered remotely and intended to increase prospection, decrease DD and promote cessation are Episodic Future Thinking (EFT) and Future Thinking Priming (FTP). EFT and FTP have demonstrated at least modest reductions in delay discounting, but understanding whether these interventions are internally valid (i.e., are accomplishing the stated intention) is key. This study examined the internal validity of EFT and FTP. Participants (*n* = 20) seeking to quit smoking were randomly assigned to active or control conditions of EFT and FTP. Linguistic Inquiry Word Count (LIWC2015) was used to examine the language participants used while engaged in the tasks. Results revealed significant differences in the language participants used in the active and control conditions. Women employed more words than men, but no other demographic differences were found in language. The active conditions for both tasks showed a greater emphasis on future orientation. Risk-avoidance was significantly higher in the active vs. control condition for EFT. Remote delivery of both EFT and FTP was valid and feasible as participants adhered to instructions in the remote prompts, and trends in DD were in the expected directions.

## 1. Introduction

Smoking tobacco remains one of the greatest preventable causes of death and disease today [1]. Most cigarette smokers express the desire to stop smoking and over half attempt to quit each year [2]. Unfortunately, more than 90% relapse within 12 months [2,3], choosing the immediate satisfaction from smoking over the temporally distant benefits and rewards achieved from not smoking. The conundrum of continued smoking despite the expressed and demonstrated desire to quit continues to be a significant public health challenge.

Although benefits and rewards lose perceived value the longer we wait to receive them, there is considerable variability in the temporal window within which we make these determinations. The degree to which one discounts or devalues delayed reinforcers is called the delay discounting (DD) rate [4,5,6,7]. DD rates are reliably associated with many aspects of cigarette smoking. Cigarette smokers show higher DD rates than non-smokers; DD rates decrease when smokers quit or reduce consumption; and DD rates reliably predict relapse after treatment for smoking cessation. DD rates have strong associations with smoking status, response to treatments for tobacco dependence, and relapse to smoking (i.e., higher baseline DD is associated with a greater propensity to relapse). DD rates can also be altered, at least temporarily, with a variety of methods [8,9,10,11,12]. DD rates, reflective of the temporal window within which rewards are valued, have become a new therapeutic target in smoking cessation treatment.

Episodic Future Thinking (EFT) and Future Thinking Priming (FTP) are two methods that have been shown to reduce proximal DD rates [13,14]. Both methods propose to alter temporal orientation by encouraging a cognitive bias toward “future thinking”, or prospection, thereby widening the temporal window to support increased value placed on temporally distant rewards. EFT involves exposing individuals to positive, anticipated, future autobiographical events via written or auditory prompts. EFT has been shown to activate brain regions involved in future thinking, planning, and executive function [15,16] to significantly decrease cigarette consumption in laboratory settings [17,18] and to reduce calorie consumption among obese individuals [19,20,21,22] (but see [23]). To develop active EFT stimuli, participants most often engage in a preliminary structured interview in which they describe positive, anticipated, future autobiographical events within several temporal windows (e.g., typically 3 or more). Typical control EFT methods require participants to engage in the same process with positive events that occurred in the recent past (e.g., in the last week). Research assistants edit the transcribed content into condensed, meaningful summaries to be used as the EFT stimuli.

FTP requires individuals to use a set of specially selected future-oriented words to create self-referential language. FTP has been shown to significantly decrease proximal DD rates in large and diverse groups of remote participants [13,24] and appears to be particularly effective at reducing DD among cigarette smokers [25]. For FTP in particular, where the respondent shoulders the burden of repeatedly producing self-referential language, it is important to examine the extent to which participants’ responses are aligned with prospective thinking over time. For the FTP task, participants use the same ten future-oriented words in a self-referential sentence and paragraph. The control FTP task requires the same procedures with ten neutral words.

While findings that EFT and FTP can decrease DD shortly after exposure is compelling, evidence that the methods do, in fact, promote prospective thinking would provide a key manipulation check. At least one prior study reports a manipulation check where participants rated the frequency with which they engaged in EFT during decision-making tasks and the vividness of cues during these tasks (e.g., “how often did you…how vivid were your thoughts about…”), showing that active EFT frequency ratings were significantly higher than control [26]. EFT and FTP require the production of language about oneself. Thus, the language produced during these tasks is likely to reflect evidence of participants’ thought processes. Linguistic Inquiry and Word Count (LIWC) is a text analysis program that calculates the frequency with which specific categories of words are used in text. The LIWC program assessed: (1) Temporal orientation—the percentage of words in past, present, and future tenses; (2) Affect—the percentage of words that indicate positive (e.g., love, nice, sweet) and negative (e.g., hurt, ugly, nasty) affect; and (3) Core drives which include the percentage of words that indicate risk-avoidance (e.g., danger, concerns, doubt) and reward-seeking (e.g., benefits, goals). Summary variables include: (1) Analytic (i.e., the degree to which the text reflects formal, logical, or hierarchical thinking); (2) Clout (i.e., expertise and confidence); (3) Authentic (i.e., honest, personal, or disclosing text); and (4) Emotional tone (i.e., text that reflects a scale from negativity (e.g., sadness, anxiety, hostility) to positivity (e.g., upbeat)). The LIWC has been extensively used in research to examine these themes, which are not often readily apparent in language and text.

This study used LIWC to examine the language participants produced as they developed EFT stimuli and when they completed the FTP tasks. Data were collected as part of a pilot study that examined the feasibility of remotely administered EFT and FTP among cigarette smokers who contacted the New York State Smokers’ Quitline (NYSSQL) for cessation assistance. Our aim was to provide an overall characterization of the language used in the active and control tasks, examine demographic differences, and test three hypotheses:

**Hypothesis** **1.** *Active EFT and FTP conditions will produce language with significantly more future orientation than EFT and FTP control conditions. Likewise, the EFT control condition will produce language with significantly more past orientation than the EFT active condition. Support for Hypothesis 1 would validate that active conditions influence prospection more than control conditions.*

**Hypothesis** **2.** *Active FTP will produce more language characteristics associated with successful goal-attainment (e.g., greater risk-avoidance, reward, positive affect, etc.) than FTP control tasks. Support for Hypothesis 2 would validate that producing future-oriented self-referential statements promotes features of successful goal-attainment.*

**Hypothesis** **3.** *The linguistic characteristics of the raw EFT transcriptions will be significantly correlated with the EFT summaries generated by research assistants. Support for Hypothesis 3 would validate that researchers are not significantly altering the content of participants’ described events by consolidating them into succinct event cues.*

## 2. Materials and Methods

### 2.1. Participants

Participants were cigarette smokers who sought treatment from the NYSSQL to quit smoking. Eligibility criteria was assessed as part of standard intake for the NYSSQL and interested individuals were contacted by the study team to complete baseline assessments. Inclusion criteria were ≥18 years old, smoking ≥8 cigarettes per day [27], no regular use of other tobacco products, willingness to quit smoking in the next 14 days, not currently using bupropion or varenicline, consuming <20 alcoholic drinks per week, and no use of drugs of abuse (e.g., cocaine, heroin, cannabis) in the past 30 days. In the parent study, the main outcome is tobacco cessation so participants who were using other tobacco products, cessation medication, or who reported co-use of other substances were excluded. Participants were also required to have regular access to the internet and an email address accessible at least every other day. Only one participant per household was allowed to enroll. All participants provided informed consent. The protocol was approved by the Roswell Park Institutional Review Board.

### 2.2. Design & Procedures

The parent study employed a fully crossed 2 × 2 randomized factorial design with two factors (EFT and FTP), each with two levels (active and control) resulting in four conditions (Active EFT/Active FTP, Active EFT/Control FTP, Control EFT/Active FTP, Control EFT/Control FTP). Participants were randomized using permuted block randomization stratified by high or low nicotine dependence level assessed with the Fagerström Test for Nicotine Dependence (FTND; high (≥4) or low (<4)). The study was conducted remotely by telephone and used Qualtrics, a web-based data collection management platform. All participants completed the baseline assessment online and then the EFT active or control interviews over the phone which were then transcribed. EFT stimuli, a more concise version of the interview, were developed accordingly, using the transcriptions of the interviews. EFT or FTP tasks were assigned on alternating weeks for a total of six administrations of each task. To control for order effects, the order in which the EFT or FTP tasks were initially presented were counterbalanced. Participants were required to complete each task within 72 h. Every two weeks, participants completed a timeline follow-back procedure reporting the number of cigarettes smoked each day for the 14 previous days. Four- and 12-weeks after initiating the tasks, participants completed outcome assessments. Participants received $20 for the baseline assessment, $10 for each TLFB assessment, and $10 for the outcome assessment. Compensation was in the form of a check. All participants received smoking cessation treatment by telephone as usual from the NYSSQL. Quitline coaches were unaware of participants’ study participation.

#### 2.2.1. Episodic Future Thinking

A specially trained research assistant engaged participants in a structured telephone interview to develop EFT stimuli. Active EFT participants were asked to describe positive, autobiographical events 6 months, 1 year, and 2 years into the future. Control EFT participants were asked to describe three positive, autobiographical events that occurred in the past 7 days. During the interview, participants were asked probing questions about the event (e.g., using Who, What, When, Where, Why, and How) and to rate the vividness of the experience. If participants were unable to reach a vividness score of 4 or greater on a scale of 1 (low) to 5 (high), research staff attempted to increase vividness with additional probing. Participants who were unable to achieve vividness scores of 4 or higher were excluded. The interview was recorded, transcribed, and synthesized into brief, naturally flowing descriptions of each event retaining as much of the participants’ descriptive words and phrasing as possible. The EFT task was presented to participants via a link in an email that, once opened, remotely guided participants through imagining each event as well as an assessment of the vividness of the experience.

#### 2.2.2. Future Thinking Priming

FTP used the same 10 words for all participants in each exposure. The FTP task was presented to participants via a link in an email that, once opened, remotely guided participants through the task as follows: Participants were presented with 10 active or control words and prompted to generate 10 different self-referential sentences, one for each word. The Active FTP words were: future, self-discipline, willpower, discipline, restraint, self-control, long-term, save, planned, and investment. The Control FTP words were: pale, drab, informative, patriotic, detached, dispassionate, middle of the road, disinterested, loud, and formal. Participants were then asked to write a short self-referential paragraph using all 10 of the words provided. Lastly, participants were asked to rate the degree to which they identified with the paragraph on a scale from 1 (low) to 5 (high). To assess compliance, the FTP sentences and paragraphs were scored for completion in line with our previous work [24]. For each FTP task, 1 point was awarded for each word used correctly in a self-referential sentence and 1 point was awarded for using each word correctly in the self-referential paragraph, for a total of 20 possible points. Each administration was scored as a percentage of points awarded over the total possible points.

### 2.3. Measures

Demographic characteristics, collected at baseline, included sex at birth, age, race, employment status, education level, partnered status, household income, cigarettes per day, and FTND. For each segment of text, LIWC analyzed word count and words per sentence; time orientation (past, present, and future focus); positive and negative affect; risk and reward; and four summary measures: analytic, clout, authentic, and emotional tone. Most output variables are expressed as a percentage of total words with six notable exceptions: word count (raw total), words per sentence (mean words per sentence), and the summary variables (analytic, clout, authentic, and emotional tone; rescaled to reflect a scale of 0–100). Analytic assessed the degree to which the text reflected formal, logical, or hierarchical thinking. Clout assessed the degree to which the text reflected expertise and confidence. Authentic assessed the presence of honest, personal, or disclosing text. Emotional tone was scored from 0–100 with higher numbers reflecting a more positive, upbeat style and lower numbers reflecting greater anxiety, sadness, or hostility. An emotional tone around 50 suggests either a lack of emotionality or ambivalence.

### 2.4. Data Analysis

The EFT stimuli and the six FTP administrations were prepared for analysis by eliminating interviewer prompts (EFT only), removing filler words and statements (e.g., “um”, “er”, “uh”, “I mean”, “You know”, “I don’t know”, etc.), and correcting grammar and spelling. The EFT text was entered in three ways: EFT-Full Raw which included all three events in the participants’ own words from the transcription; EFT-Events Raw which included three separate paragraphs, one for each event in the participants’ own words from the transcription; and EFT-Brief which included three separate summary paragraphs, one for each event developed by research assistants. The FTP text for the sentences and the associated paragraph was entered once for each administration, for a possible total of six FTP texts for each participant.

The text produced in EFT and FTP were analyzed using LIWC2015 (Pennebaker Conglomerates Inc., Austin, TX, USA). Due to unequal attrition between active and control FTP conditions over time, linguistic values derived from the LIWC2015 for each administration of the FTP task were averaged by adding values and dividing by the number of administrations for each participant. Independent samples t-tests were performed to examine differences between the active and control conditions for EFT-Full Raw and FTP for raw word count and mean words per sentence. Independent samples t-tests were used to examine demographic differences for raw word count and mean words per sentence.

Independent samples t-tests were used to examine differences between the active and control conditions for EFT-Full Raw and FTP on time perspective, with particular attention to future orientation. Independent samples t-tests were also used to examine differences in goal-attainment language (e.g., positive and negative affect, core drives of risk and reward, etc.) between active and control FTP. Bivariate Pearson correlations were used to examine correlations between EFT-Events Raw (the raw transcript for each event) and EFT-Brief (the summary of each event developed by the researchers). To summarize bivariate Pearson correlations between the EFT-Events Raw and EFT-Brief values, a correlation matrix was generated for each event paragraph (1, 2, and 3) of the EFT. Then, correlation coefficients were translated to z-scores, averaged, and the average was re-converted to a correlation coefficient. While all data met the assumptions for parametric testing, non-parametric tests were performed as well due to small sample size. Results where the testing modality changed the significance in either direction are presented. All analyses were conducted using IBM SPSS, Version 23.

## 3. Results

Participants’ demographic characteristics are presented in Table 1. Participants (*n* = 20) were 65% women, 50% white, and 75% unpartnered. Half (50%) had annual household incomes of <$25,000, and 40% had a high school education or less. Participants smoked, on average, 15.2 cigarettes per day, and were moderately nicotine dependent, scoring a mean of 4.8 on the FTND. Participants largely complied with the instructions for FTP; the mean compliance rate was 82.9% (SD = 19.8%).

The mean word count for EFT-Full Raw was 1401.1 (869.2), with a mean of 11.9 (3.3) words per sentence. The mean word count for the EFT-Brief was 368.3 (114.9), and for EFT-Events Raw it was 1167.7 (689.4). The mean word count across all FTP administrations was 186.4 (48.5). There were no significant differences in mean word count [1453.1(1100.5) vs. 1373.0(766.9); *p* = 0.85] or mean words per sentence [10.2(2.2) vs. (12.9(3.5)); *p* = 0.08] between the active and control conditions for EFT-Full Raw. Similarly, there were no significant differences between active and control conditions of FTP for mean word count [186.3(43.6) vs. 186.4(54.9); *p* = 0.99] or mean words per sentence [12.2(2.6) vs. 11.7(3.6); *p* = 0.73].

Women produced significantly more words per sentence in EFT-Full Raw than men [13.10(3.1) vs. 9.88(2.7); *p* = 0.03 [−6.1–−0.29]], but no sex differences in word count were found [*p* = 0.81]. No sex differences were found for FTP in word count [*p* = 0.99] or words per sentence [*p* = 0.94]. Age was not significantly correlated with word count or words per sentence for EFT-Full Raw or FTP [p’s > 0.29]. No differences in word count or words per sentence were found for race, employment, or education status in EFT-Full Raw or FTP [p’s > 0.38]. Partnered individuals had a greater word count on EFT-Full Raw than unpartnered individuals [2203.0(1022.7) vs. 1133.7(648.3); *p* = 0.01 [−1880.6–257.9]. This difference was not further explained by an interaction with sex. No partner-related difference in word count was found for FTP [*p* = 0.84]. No partner-related differences were found on words per sentence for EFT-Full Raw [*p* = 0.64] or FTP [*p* = 0.91]. No difference in word count or words per sentence were found in household income in EFT-Full Raw or FTP [p’s > 0.69]. Cigarettes per day and FTND were not significantly correlated with word count or words per sentence for either task [p’s > 0.07].

Active EFT-Full Raw showed significantly lower clout than the control condition [30.9(11.1) vs. 49.8(19.8); *p* = 0.03 [1.7–35.9]]. No significant differences were found in analytic or authentic text between active and control conditions for EFT-Full Raw [*p* = 0.47; *p* = 0.26]. No significant differences were found in analytic, clout, or authentic text between active and control conditions for FTP [*p* = 0.25; *p* = 0.06; *p* = 0.09].

**Figure 1:**
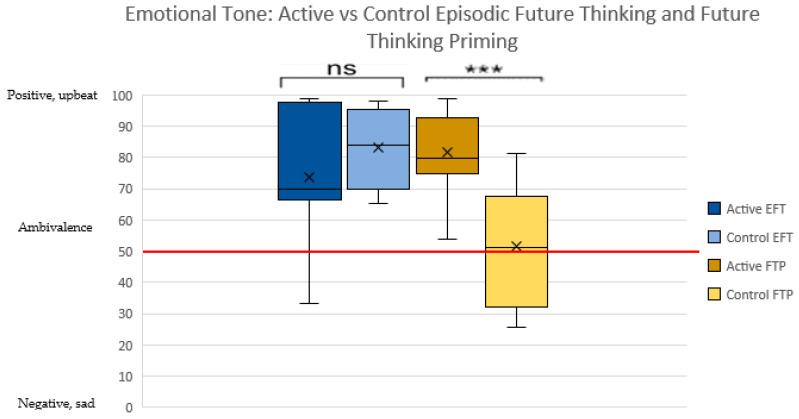
Emotional tone by Episodic Future Thinking and Future Thinking Priming active vs. control conditions. Emotional tone, a scale from 0–100, reflects a spectrum from negative, sad, anxious, or hostile (score of 0) to positive and upbeat (score of 100). Scores around 50, denoted by the red line, indicate ambivalence. Note: *** = *p* < 0.001, ns = not significant.

The FTP active condition demonstrated a more positive, upbeat tone than the control condition, which was generally ambivalent [81.65(13.5) vs. (51.6(18.8); *p* = 0.001 [−46.1—14.1]]. However, no significant differences were found in emotional tone between active and control conditions for EFT-Full Raw [*p* = 0.23] (see Figure 1).

**Figure 2:**
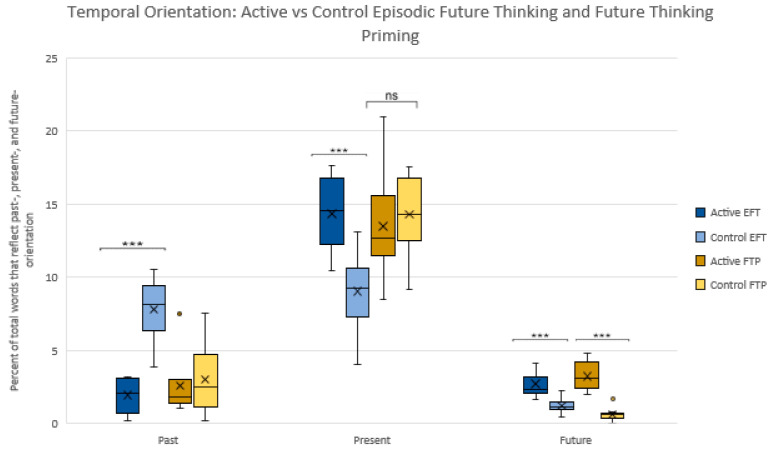
Percentage of total words that reflect past-, present-, and future-oriented text for Episodic Future Thinking and Future Thinking Priming active vs. control conditions. *** = *p* < 0.001, ns = not significant.

Hypothesis 1 was supported: The active conditions for both tasks showed a greater emphasis on future orientation, as expected. Future time perspective was significantly higher in the active vs. control conditions for EFT-Full Raw [2.71(0.83) vs. 1.23(0.49); *p* = 0.000 [−2.1–−0.87] and FTP [3.2(0.99) vs. 0.61(0.45); *p* = 0.000 [−3.3—1.9]. The control condition for EFT-Full Raw showed greater emphasis on the past compared to active [1.9(1.2) vs. 7.8(1.9); *p* = 0.000 [4.1–7.6]. No difference was found in past orientation for FTP active vs. control conditions [*p* = 0.65]. The active condition for EFT-Full Raw showed greater emphasis on present orientation compared to control [14.35(2.5) vs. 9.0(2.3); *p* = 0.000 [−7.6-−2.9. No significant difference was found in present orientation for FTP active vs. control conditions. See Figure 2.

**Figure 3:**
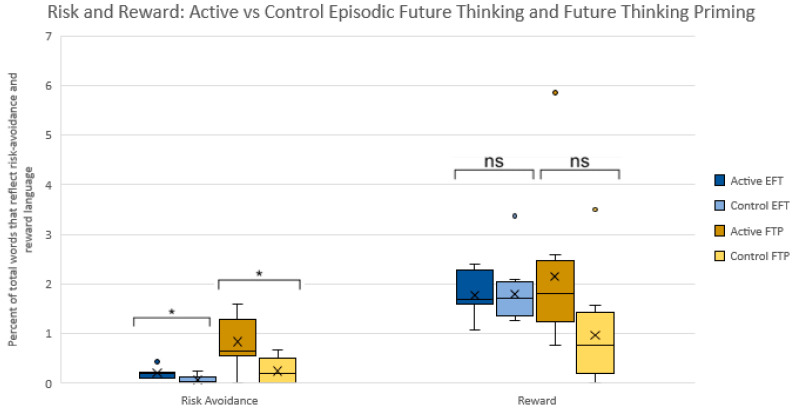
Percentage of total words that reflect risk-avoidance and reward text for Episodic Future Thinking and Future Thinking Priming active vs. control conditions. Note: * = *p* < 0.01, ns = not significant.

Hypothesis 2 was partially supported: Risk-avoidance was significantly higher in the active vs. control condition for EFT-Full Raw [0.20(0.12) vs. 0.06(0.08); *p* = 0.006 [−0.22–−0.04] and FTP [0.83(0.51) vs. 0.24(0.24); *p* = 0.004 [−0.96–−0.21]. No significant difference was found for reward focus between active and control conditions for EFT-Full Raw [*p* = 0.93] Reward focus was higher in active FTP conditions, but only approached significance compared to control [2.16(1.51) vs. 0.96(1.04); *p* = 0.06]. A Mann-Whitney test, however, indicated that this difference was statistically significant, *U*(*N_active_* = 9, *N*_control_ = 10) = 74.00, *z* = 2.37, *p* = 0.02 See Figure 3.

**Figure 4:**
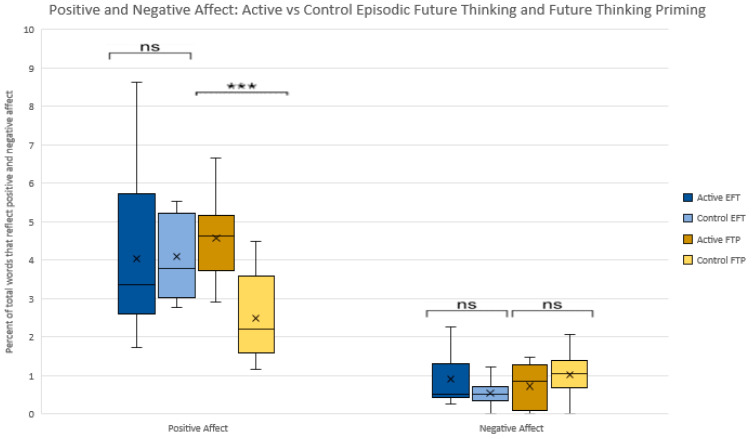
Percentage of total words that reflect positive and negative affect for Episodic Future Thinking and Future Thinking Priming active vs. control conditions. *** = *p* < 0.001, ns = not significant.

No significant differences were found for positive or negative affect between active and control conditions for EFT-Full Raw [*p* = 0.95; *p* = 0.14]. No significant differences were found for negative affect between active and control conditions for FTP [*p* = 0.26]. Positive affect was significantly higher in the active vs. control conditions for FTP [4.57(1.11) vs. 2.49(1.11); *p* = 0.001 [−3.2–1.0]. See Figure 4. Interestingly, follow-up exploratory analyses found positive affect to be significantly correlated with future time perspective [r = 0.51, *p* = 0.03] in FTP but not in EFT [r = −0.09, *p* = 0.70]. Conversely, negative affect was significantly correlated with future time perspective [r = 0.51, *p* = 0.02] in EFT, but not in FTP [r = −0.31, *p* = 0.19]. For EFT and FTP, as emotional tone increased, positive affect increased [r = 0.84, *p* = 0.000; r = 0.93, *p* = 0.000]. For FTP, as emotional tone decreased, negative affect increased [r = −0.56, *p* = 0.012], consistent with what might be expected. For EFT, emotional tone and negative affect were not significantly correlated [r = −0.20, *p* = 0.40].

Hypothesis 3 was supported. The EFT-Brief summaries were significantly correlated with the EFT-Events Raw text for all linguistic analysis variables with the exception of Words per Sentence, Risk, and Analytic. See Table 2.

**Figure 5:**
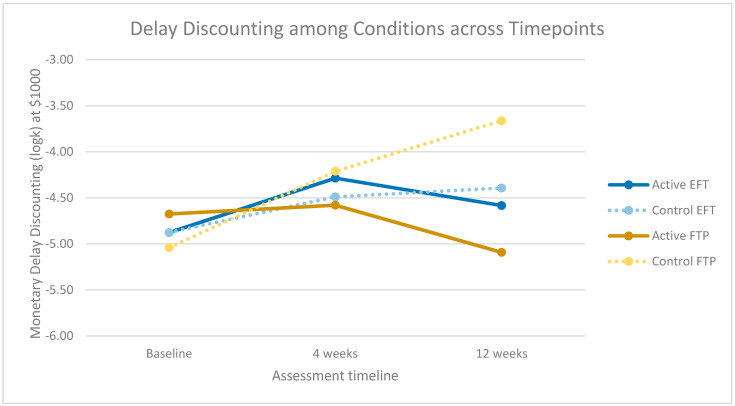
Monetary delay discounting using Mazur’s k hyperbolic equation at the $1000 amount for active and control EFT and FTP conditions from baseline through 12 weeks.

Spearman’s rank correlations revealed different results only in words per sentence, which became significant (ρ = 0.69, *p* = 0.00) and reward, which no longer showed significance (ρ = 0.26, *p* = 0.26). The study was underpowered to detect statistical differences in DD among groups [28], largely due to the SARS-CoV-2 pandemic. However, Figure 5 shows that trends are in the expected direction such that control conditions for both EFT and FTP consistently trend upwards and active EFT and FTP show a downward shift around 12 weeks.

## 4. Discussion

Overall, EFT and FTP, two promising methods for altering temporal orientation, show evidence for strong internal validity. The language used by participants assigned to the active conditions for both methods showed greater future orientation than the language used in the control conditions, indicating that both experimental manipulations support prospection. In addition, the EFT control condition showed greater past orientation consistent with the intent of the instructions. The EFT active condition showed greater present focus compared to control which may be related to the English use of the present participle when constructing future sentences (e.g., “I will be going.”). No differences in word count or words per sentence were found between the active and control conditions for either intervention indicating that active effects are unlikely to be caused by increased engagement. FTP demonstrated no differences between the active and control conditions in analytic, clout, and authentic text indicating that the active effects of FTP are also unlikely to be caused by differences in these qualities. For EFT, there were no differences in analytic and authentic text between active and control conditions. The EFT control condition showed significantly greater clout than the active condition indicating that participants were more confident about events in the past 7 days than in the next 6 months which is something to be considered while developing EFT stimuli in the future. For instance, increasing clout in the active EFT condition might increase the impact of the intervention on prospection. Altogether, these findings suggest that individuals in active and control conditions for EFT and FTP did not significantly differ in levels of participation and task engagement; as a result, differences in our target variables may reasonably be assumed to be related to increased prospection.

Prospection during decision making is inextricably linked with perceived reward, risk, and affect [29]. Evidence for the internal validity of the EFT and FTP manipulation might also be interpreted from other characteristics of the language used in these methods to increase prospection. Consistent with increased prospection, the active conditions in both methods demonstrated greater risk avoidance. Increased risk-prevention focus has been significantly related to positive health behavior change as well, even when holding factors such as optimism constant [30]. Reward focus was not significantly different between the active and the control conditions for either method, although a greater focus on reward approached significance for FTP. Increasing the reward focus in the language used in EFT and FTP is again something to be considered for the future.

Positive and negative affect can impact prospection [31]. Greater indications of positive affect are significant because increasing positive affect has been suggested as an underlying framework for improving incentive salience, thereby promoting positive health behavior change [32]. In this study, for EFT, the level of positive and negative affect did not differ between active and control conditions suggesting that affect was held constant between the two EFT conditions, consistent with the instructions. The FTP had no instruction to produce positive language for either the active or the control conditions. For FTP, the active condition produced significantly greater positive affect than control but negative affect did not differ. In the absence of instructions to produce positive language, it appears that encouraging prospection with FTP promotes positive affect.

Finally, these findings indicate that researcher summaries shortened the text but did not meaningfully alter the linguistic content of the original EFT interviews (see Table 2). Risk-avoidance in the EFT-Brief may not have been correlated with the EFT-Events Raw because researchers might have selected or created more confident statements in their summaries. Reticent sentences in the full transcript may have been both more revealing of risk (i.e., danger, concerns, and things to avoid) and more likely to be excluded. Analytic text was greater in the EFT-Brief than the EFT-Events Raw. Higher scores on analytic text are related to greater formal, logical, and hierarchical organization—something researchers might reasonably have been expected to produce. Lower analytic scores in the EFT-Events Raw might be related to the informal nature of the phone interview. These findings should be considered in developing EFT stimuli in the future.

These two promising methods might be well-suited for the diverse populations served by Quitlines. The participants in this study were diverse in terms of sex, race, and socioeconomic status. The consistency in word count and words per sentence among these demographic factors suggests that EFT and FTP are both unlikely to produce racial or socioeconomic biases in engagement. Although women produced more words per sentence and perhaps more complex sentences, they did not produce more words overall. In addition, cigarettes per day and level of dependence appeared to have no relation to word count or words per sentence suggesting that these factors are also unlikely to impact engagement. Remotely delivered EFT and FTP among Quitline callers seeking smoking cessation assistance might provide an opportunity to increase engagement with Quitline services, improve the efficacy of current treatments, and/or increase the reach to populations who are unwilling or unable to use other treatment modalities, but more research is needed to examine the impact of these methods on cessation. Future studies should ensure timely payment for research tasks to enhance retention and a larger number of participants (using the more conservative estimated effect size of FTP, an estimated 1000 participants, 250 per condition, may be suggested) [28].

This novel approach to testing internal validity for language-based interventions to increase prospection had a few limitations and several strengths. Limitations include a small sample size and relatively high study attrition. We believe attrition is largely due to an untimely compensation process (mailing checks after task completion) and the impacts of the SARS-CoV−2 pandemic. Though the pilot study was paused due to the SARS-CoV-2 pandemic, and therefore underpowered to detect significant changes in DD, available data were plotted to examine trends across groups. The graphic representation of changes in DD provided a visual indication of internal validity but was not a primary outcome of this investigation. Along with a small sample size, this study ran the risk for over-testing, though the outcome measures were each assessed only once, and therefore were not subject to limitations relative to multiple comparisons.

Strengths include the diversity of the sample; the innovative nature of using language as evidence of prospection; analyzing the linguistic content of EFT in 3 ways; and an easily replicable design focusing on remote delivery.

## 5. Conclusions

The findings of this small-scale pilot study are encouraging in that participants in this remotely conducted study took the assigned tasks seriously, regardless of active or control assignment; the experimental manipulation was successful; and there was little evidence of a reduction in text quality over time. Both EFT and FTP can be remotely delivered, providing a low-cost, high-reach alternative to one-on-one counseling sessions which can easily be applied in Quitline settings.

Future research should apply text analysis to broader samples of EFT and/or FTP writings to determine whether the patterns observed here are consistent in larger, more diverse groups, and across health behavior domains. Additionally, our small sample exhibited some interesting demographic trends related to word count and words per sentence; these should be investigated in larger and more diverse samples.

Text-intensive interventions such as EFT and FTP have demonstrated efficacy in altering DD, the manipulation of which is linked to improving health behaviors. The current study, part of a pilot examination of EFT and FTP interventions for smoking cessation, exhibited high compliance with task instructions and the expected trends in DD despite being underpowered. The overall content of writing for both EFT and FTP (control and active) was comparable, with negligible differences in word count. Active EFT and FTP produced the expected changes in prospection as well as linguistic characteristics related to goal-attainment. This supports the feasibility of conducting large scale, remote studies that successfully alter temporal orientation among cigarette smokers.

## Figures and Tables

**Table 1:** Baseline demographic characteristics.

Variable	Categories or Ranges	% (*n*) or Mean (SD)
Sex	Female	65% (13)
Age	26–68	57.5% (10.6)
Race	White	50% (10)
	Black	30% (6)
	Multi-ethnic or Other	20% (4)
Employment	Employed full-time	40% (8)
	Disabled	20% (4)
	Unemployed	20% (4)
	Retired	15% (3)
	Employed part-time	5% (1)
Education	High school or less	40% (8)
	College or trade school	55% (11)
	Graduate school	5% (1)
Partnered Status	Unpartnered	75% (15)
Household Income	<$25,000	50% (10)
	$25,000–$49,999	20% (4)
	>$50,000	30% (6)
Cigarettes per Day	8–40	15.2 (8.6)
FTND ^1^	0–10	4.8 (2.3)

^1^ FTND = Fagerström Test for Nicotine Dependence.

**Table 2:** Correlation matrix for EFT-Events Raw vs. EFT-Events Brief linguistic variables.

Linguistic Variable	Pearson *r*[CI]
Word Count	0.70 *** [0.69–0.71]
Words per Sentence	0.43 [0.42–0.44]
Past Orientation	0.99 *** [0.99–0.99]
Present Orientation	0.69 *** [0.68–0.69]
Future Orientation	0.65 ** [0.64–0.66]
Positive Affect	0.64 ** [0.63–0.65]
Negative Affect	0.65 ** [0.64–0.66]
Risk-avoidance	0.36 [0.35–0.37]
Reward	0.46 * [0.45–0.47]
Analytic	0.31 [0.29–0.32]
Clout	0.84 *** [0.84–0.84]
Authentic	0.59 ** [0.58–0.60]
Emotional Tone	0.50 * [0.49–0.51]

* *p* < 0.05; ** *p* < 0.01; *** *p* < 0.001.

## Data Availability

The data presented in this study are available on request from the corresponding author. The data are not publicly available to retain participant privacy.
